# Radiomics Metrics Combined with Clinical Data in the Surgical Management of Early-Stage (cT1–T2 N0) Tongue Squamous Cell Carcinomas: A Preliminary Study

**DOI:** 10.3390/biology11030468

**Published:** 2022-03-18

**Authors:** Umberto Committeri, Roberta Fusco, Elio Di Bernardo, Vincenzo Abbate, Giovanni Salzano, Fabio Maglitto, Giovanni Dell’Aversana Orabona, Pasquale Piombino, Paola Bonavolontà, Antonio Arena, Francesco Perri, Maria Grazia Maglione, Sergio Venanzio Setola, Vincenza Granata, Giorgio Iaconetta, Franco Ionna, Antonella Petrillo, Luigi Califano

**Affiliations:** 1 Maxillofacial Surgery Operative Unit, Department of Neurosciences, Reproductive and Odontostomatological Sciences, Federico II University of Naples, 80131 Naples, Italy; umbertocommitteri@gmail.com (U.C.); vincenzo.abbate@unina.it (V.A.); giovannisalzanomd@gmail.com (G.S.); fabio.maglitto@unina.it (F.M.); giovanni.dellaversanaorabona@unina.it (G.D.O.); pasquale.piombino@unina.it (P.P.); paola.bonavolonta@unina.it (P.B.); antonio.arena@unina.it (A.A.); luigi.califano@unina.it (L.C.); 2 Medical Oncology Division, Igea SpA, 80013 Naples, Italy; r.fusco@igeamedical.com (R.F.); e.dibernardo@igeamedical.com (E.D.B.); 3 Head and Neck Medical Oncology Unit, Istituto Nazionale dei Tumori IRCCS Fondazione G. Pascale, 80131 Naples, Italy; f.perri@istitutotumori.na.it; 4 Division of Surgical Oncology Maxillo-Facial Unit, Istituto Nazionale Tumori-IRCCS-Fondazione G. Pascale, Via Mariano Semmola, 80131 Naples, Italy; m.maglione@istitutotumori.na.it (M.G.M.); f.ionna@istitutotumori.na.it (F.I.); 5 Divisions of Radiology, Istituto Nazionale dei Tumori IRCCS Fondazione G. Pascale, 80131 Naples, Italy; s.setola@istitutotumori.na.it (S.V.S.); v.granata@istitutotumori.na.it (V.G.); 6 Department of Neurosurgery, University of Salerno, 84084 Salerno, Italy; giaconetta@unisa.it

**Keywords:** radiomics, machine learning, oral tongue squamous cell carcinoma (OTSCC), neck dissection, inflammatory index, depth of invasion (DOI)

## Abstract

**Simple Summary:**

The purpose of this study is to predict the risk of metastatic lymph nodes related to oral tongue squamous cell carcinoma (OTSCC) and tumor grading through the combination of clinical data with radiomics metrics extracted from computed tomography (CT) images. We aimed to develop a supportive approach in the management of lymphatic cervical areas, with particular attention given to the early stages (T1−T2). Specifically, we evaluated the effectiveness of the radiomics and clinical features in the study of OTSCC and in the prediction of occult laterocervical metastatic lymph nodes. We concluded that radiomics features and clinical parameters have an important role in identifying tumor grading and metastatic lymph nodes. Machine learning approaches can be used as an easy-to-use tool to stratify patients with early-stage OTSCC, based on the identification of metastatic and non-metastatic lymph nodes

**Abstract:**

Objective: To predict the risk of metastatic lymph nodes and the tumor grading related to oral tongue squamous cell carcinoma (OTSCC) through the combination of clinical data with radiomics metrics by computed tomography, and to develop a supportive approach in the management of the lymphatic cervical areas, with particular attention to the early stages (T1−T2). Between March 2016 and February 2020, patients with histologically confirmed OTSCC, treated by partial glossectomy and ipsilateral laterocervical lymphadenectomy and subjected to computed tomography (CT) before surgery, were identified by two centers: 81 patients (49 female and 32 male) with 58 years as the median age (range 19–86 years). Univariate analysis with non-parametric tests and multivariate analysis with machine learning approaches were used. Clinical, hematological parameters and radiological features extracted by CT were considered individually and in combination. All clinical parameters showed statistically significant differences (*p* < 0.05) for the Kruskal−Wallis test when discriminating both the tumor grading and the metastatic lymph nodes. DOI, PLR, SII, and SIRI showed an accuracy of 0.70 (ROC analysis) when identifying the tumor grading, while an accuracy ≥ 0.78 was shown by DOI, NLR, PLR, SII, and SIRI when discriminating metastatic lymph nodes. In the context of the analysis of radiomics metrics, the *original_glszm_HighGrayLevelZoneEmphasis* feature was selected for identifying the tumor grading (accuracy of 0.70), while the *wavelet_HHH_glrlm_LowGrayLevelRunEmphasis* predictor was selected for determining metastatic lymph nodes (accuracy of 0.96). Remarkable findings were also obtained when classifying patients with a machine learning approach. Radiomics features alone can predict tumor grading with an accuracy of 0.76 using a logistic regression model, while an accuracy of 0.82 can be obtained by running a CART algorithm through a combination of three clinical parameters (SIRI, DOI, and PLR) with a radiomics feature (*wavelet_LLL_glszm_SizeZoneNonUniformityNormalized*). In the context of predicting metastatic lymph nodes, an accuracy of 0.94 was obtained using 15 radiomics features in a logistic regression model, while both CART and CIDT achieved an asymptotic accuracy value of 1.00 using only one radiomics feature. Radiomics features and clinical parameters have an important role in identifying tumor grading and metastatic lymph nodes. Machine learning approaches can be used as an easy-to-use tool to stratify patients with early-stage OTSCC, based on the identification of metastatic and non-metastatic lymph nodes.

## 1. Introduction

Oral tongue squamous cell carcinoma (OTSCC) represents an aggressive tumor associated with a poor prognosis due to the high risk of local and locoregional invasiveness [1].

Tongue drainage involves nodes of I, II, and partially III level of the lymphatic system of the neck. About 35% of patients in stage I–II of oral cancer have a rather high risk of developing occult cervical metastases, with a significant reduction of survival rate of 50% [2]. 

The management of the neck lymphatic stations in T1−T2 CN0 OTSCC, indeed, represents a dilemma and the surgical decision is often based on individual clinical experience. Nowadays, the surgeon can opt for several therapeutic alternatives. In patients with early-stage tumors, a watchful waiting approach can be adopted, otherwise elective neck dissection or biopsy of sentinel lymph node have to be considered. In this regard, it is important to note that elective neck dissection is not free from complications (rate close to 29%); this approach, in fact, is burdened by greater morbidity, given by the second surgical site. In 70–80% of cases, this option can represent overtreatment [3]. Nevertheless, the sentinel lymph node biopsy has a sensitivity that varies in the literature between 75−100%, for identifying the first draining lymph node in squamous carcinomas of the oral cavity; there is also a not negligible percentage of false negatives of about 14% [4].

The eighth edition of the American Joint Commission on Cancer (AJCC) provides, as a novelty in the TNM classification of oral cavity carcinomas, the depth of invasion (DOI), which evaluates the primary tumor (T), drawing a perpendicular line passing through the basement membrane of the normal epithelium, contiguous to the neoplasm, up to the deepest tumor invasion point. It seems with a DOI cut-off between 3 and 4 mm there is a 20% risk of lymphatic micro metastases [5]. This condition could be a possible recommendation for elective neck levels I-I-II, which overrides the extension to IV if the tumor has a posterior origin.

Furthermore, the state of inflammation also has a relationship with cancerogenesis. Rudolf Virchow described for the first time a possible association between the state of inflammation and cancer [6]. However, only in the last few decades has a clear relationship between inflammation and carcinogenesis been established [6], thanks to the possibility of detecting the systemic inflammatory state through blood samples, namely through the increase in the neutrophil, monocyte, or platelet count.

In addition, an association between the inflammation indices and the possible decreased survival in different tumor models was found, including patients with squamous cell carcinomas of the head−neck area. On the other hand, the presence of lymphopenia reflects a possible reduction in immune response with an aggravation of the prognosis.

Recently, all these hematological parameters, indicative of a state inflammatory and/or immune failure, have been combined for obtaining new prognostic biomarkers. Among these, the best known are the neutrophils−lymphocytes ratio (NLR); the platelet−lymphocyte ratio (PLR); the lymphocytes−monocytes ratio (LMR); the Systemic Inflammation Response Index (SIRI), defined as neutrophil count × monocyte/lymphocyte count; and the Systemic Immune-inflammation Index (SII), calculated by the formula neutrophil × platelet/lymphocyte [7].

Biomarkers can be derived from a wide variety of diagnostic sources, such as from the biopsy of tissue specimens, from cell collections, and from radiological images. In the context of cancer research, particular attention has been increasingly paid to the quantitative analysis of high-performance images through radiomics. Radiomics extracts quantitative features from medical imaging with the aim of obtaining descriptors of the image contents. Moreover, these features can be combined with other characteristics of the patient, when available, for increasing the power of the decision support models. This can be performed with the appropriate use of techniques from machine learning and artificial intelligence areas [8].

The purpose of this study is to predict the risk of metastatic lymph nodes related to OTSCC and tumor grading through the combination of clinical data with radiomics metrics extracted from computed tomography (CT) images. By means of statistical analysis, we aimed to develop a supportive approach in the management of the lymphatic cervical areas, with particular attention to the early stages (T1−T2). Specifically, we evaluated the effectiveness of the radiomics and clinical features in the study of OTSCC and in the prediction of occult laterocervical metastatic lymph nodes. To this aim, the features were both considered individually and combined with each other. 

## 2. Materials and Methods

### 2.1. Patient Selection

In the context of a multicenter retrospective study, 81 patients were recruited by the Maxillofacial Surgery of the Polyclinic Federico II University of Naples and Otolaryngology and the Surgery Maxillofacial of the National Cancer Institute IRCCS G. Pascale of Naples.

The study was performed in accordance with relevant guidelines and regulations; informed consent was not required by the Local Ethics Committee due to the retrospective nature of the study.

The analysis was conducted between March 2016 and February 2020. The patients (49 female and 32 male) had a median age of 58 years (range 19–86 years) and had received a diagnosis of OTSCC. The diagnosis was confirmed with histological analysis and all patients underwent CT before surgery (partial glossectomy or ipsilateral laterocervical lymphadenectomy).

Inclusion criteria were proven histology of OTSCC with a preoperative incisional biopsy, early stage cT1−T2 OTSCC, non-primary OTSCC previously treated, availability of clinical and hematological parameters and a minimum of 12 months of post-operative clinical follow-up. Exclusion criteria were radiotherapy or chemotherapy in the clinical history, previous tumor in other sites, advanced squamous cell carcinoma, chronic inflammatory pathologies capable of influencing the indices of inflammation (infections, serum virus markers, chronic or autoimmune inflammatory diseases, haematological disorders, and simultaneous or long-term anti-inflammatory or steroidal drug treatments).

Clinical and hematological parameters included: age; gender; DOI, NLR; PLR; LMR; SIRI; SII; T stage; grading; metastatic lymph nodes; perineural infiltration; vascular infiltration.

### 2.2. CT Protocol

A tomograph with 64 detectors (Optima 660, General Electric Healthcare, Milwaukee, WI, USA) was used to acquire the CT images.

The image acquisition was performed at 120–140 kVp, 200–600 mA, setting a slice thickness of 1.25–2.5 mm and a table speed/rotation of 0.938–0.984/1 mm. Head and neck contrast-enhanced CT images were acquired in the venous phase (start delay 70–80 s), after the intravenous injection of 2 mL/kg of a non-ionic contrast material (iodine concentration >350 mg/ml), followed by 40 mL of saline solution, using a semi-automated power injector (3.5–4 mL/s flow rate). 

### 2.3. Image Processing

Regions of interest (ROIs) were manually drawn, slice-by-slice, by two expert radiologists with 22 and 15 years, respectively, of experience in head and neck imaging. The radiologists performed the selection of ROIs first separately and then jointly. Manual definition of the ROIs was made using the 3DSlicer segmentation tool (https://www.slicer.org/ accessed on 15 February 2021).

In the context of radiomics, 851 features were extracted using PyRadiomics [9], in compliance with feature definitions, as described by the Imaging Biomarker Standardization Initiative (IBSI) [10]. More precisely, two sets of 851 radiomics features were extracted from both the tumor region and the lymph node area. The radiomics features were subdivided into the following classes: First order statistics, shape-based (2D and 3D); gray level cooccurence matrix (24 features), gray level run length matrix (16 features), gray level size zone matrix, neighboring gray tone difference matrix, and gray level dependence matrix. Details about radiomics features can be found in the work of Kumarasamy [9].

### 2.4. Statistical Analysis

#### 2.4.1. Univariate Analysis

The non-parametric Kruskal−Wallis test was performed to identify those features that were mostly involved in (individually) discriminating the following outcomes: G1 + G2 grading versus G3 grading, metastatic lymph nodes versus non-metastatic lymph nodes, presence or absence of perineural infiltration, and presence or absence of vascular infiltration. The receiver operating characteristic (ROC) analysis was also performed and the Youden index was used to identify the optimal cut-off value for each feature; the area under the ROC curve (AUC), sensitivity, specificity, positive predictive value (PPV), negative predictive value (NPV), and accuracy were then calculated to evaluate the performances.

The univariate analyses were performed using the Statistics Toolbox of MATLAB R2007a (MathWorks, Natick, MA, USA).

#### 2.4.2. Multivariate Analysis

A multivariate analysis was performed in order to identify the combinations of features that best predicted the outcomes: G1 + G2 grading versus G3 grading, metastatic lymph nodes versus non-metastatic lymph nodes, presence or absence of perineural infiltration, and presence or absence of vascular infiltration. 

Clinical parameters and radiomics features were considered both individually and in combination, and were extracted from both tumor and lymph node areas.

Given the high number of radiomics features, before proceeding with the analysis, a selection of variables was made on the basis of the results obtained from the univariate analysis, considering only the features that were significant in the Kruskal−Wallis test.

Three machine learning algorithms were appropriately designed and run on RStudio Version 1.3.959 (https://www.rstudio.com/ accessed on 15 February 2021), as described in the following. Here, 80% of the dataset was used to train the algorithms, while the remaining 20% was used to validate the models and compare the results. The dataset was split in a randomized way, using the *createDataPartition* R function.

Logistic Regression. Considering that all the outcomes were binary (i.e., only two values were allowed for those variables), logistic regression was executed and used as a baseline for the model comparison. Logistic regression was performed using the *glm* function on RStudio, with appropriate design of function attributes and data pre-processing (centering and scaling of input predictors).

Tree-based algorithms. Classification and Regression Trees (CART) and Conditional Inference Decision Trees (CIDT) were executed for classifying the outcome variables. The hyperparameters were tuned with a 10-fold cross validation, and were repeated 10 times. A decision chart was also obtained with the aim of supporting the surgeon in the decision process.

## 3. Results

### 3.1. Univariate Analysis

All clinical parameters showed statistically significant differences (*p* < 0.05) in the Kruskal−Wallis test on median values when discriminating both the tumor grading and the metastatic lymph nodes. 

In the context of identifying the tumor grading, DOI, PLR, SII, and SIRI showed an accuracy of 0.70 and the Systemic Immune-inflammation Index achieved the highest AUC value (0.73), with appreciable values of specificity and sensitivity (Table 1). However, better results were achieved in the ROC analysis of clinical parameters when discriminating metastatic lymph nodes. In fact, DOI, NLR, PLR, SII, and SIRI showed an accuracy ≥ 0.78 (Table 1), with PLR (accuracy of 0.83) reaching an AUC of 0.76, with sensitivity and specificity values of 0.80 and 0.85, respectively. 

Less remarkable results were found when grouping patients according to the presence/absence of both perineural and vascular infiltration. Five (DOI, PLR, LMR, SII, and SIRI) out of seven clinical parameters showed a statistically significant difference (*p* < 0.05) in the Kruskal−Wallis test of median values for evaluating the perineural infiltration, with both DOI and SII having an accuracy slightly above 0.70. DOI, NLR, SII, and SIRI were, instead, found to be statistically significant (Kruskal−Wallis test, *p* < 0.05) in discriminating the presence or the absence of vascular infiltration. In the latter case, the only variable that showed an accuracy of just 0.73 was NLR, with an AUC value of 0.64 (Table 1).

The same trend of results was also observed in the context of identifying the presence or absence of infiltration (both perineural and vascular) when performing the ROC analysis on radiomics features. As Table 2 shows, the best accuracy achievable was 0.68 with the *wavelet_HHH_glcm_MaximumProbability* feature (sensitivity of 0.69 and specificity of 0.67) related to perineural infiltration, while a slightly better result can be achieved (in term of accuracy) in the context of vascular infiltration; however, the sensitivity value was found to be significantly low (0.29). Even in the analysis conducted with the Kruskal−Wallis test, the results on both types of infiltrations were ae less noticeable; in fact, only 39 and 57 radiomics features were statistically significant for perineural and vascular infiltration, respectively.

Contrarily, the differentiation of tumor grading and metastatic lymph nodes gave more interesting results. Indeed, already in the case of the Kruskal−Wallis test, 158 out of 851 radiomics features were found to be statistically significant (*p* < 0.05) for discriminating G1 + G2 versus G3 grading, and 804 features showed an analogue significance (*p* < 0.05) when identifying metastatic from non-metastatic lymph nodes. Further remarkable results were also found in the context of the ROC analysis. The *original_glszm_HighGrayLevelZoneEmphasis* feature was selected (among the 158 significant features) given the good performance (accuracy of 0.70) achieved in identifying the tumor grading (Table 2). However, the most noteworthy results were reached when determining the metastatic lymph nodes; in fact, the *wavelet_HHH_glrlm_LowGrayLevelRunEmphasis* predictor was able to separate patients with metastatic lymph nodes from those with non-metastatic lymph nodes with an accuracy of 0.96 (sensitivity of 0.94, specificity of 0.98) and with a cut-off value equals to 0.39 (Table 2).

### 3.2. Multivariate Analysis

In the multivariate analysis, a machine learning approach was used to predict the tumor grading and the metastatic lymph nodes. The patients were classified with the algorithms and using both clinical and radiomics features, both individually and in combination. The results obtained are reported in Table 3 and Table 4, and the accuracies achieved with the logistic regression were used as a baseline.

**Figure 1:**
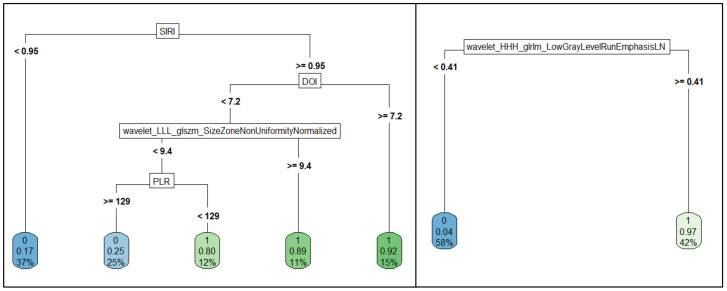
Decision trees obtained with the CART algorithm for the prediction of tumor grading (**left** panel) and metastatic lymph nodes (**right** panel).

**Figure 2:**
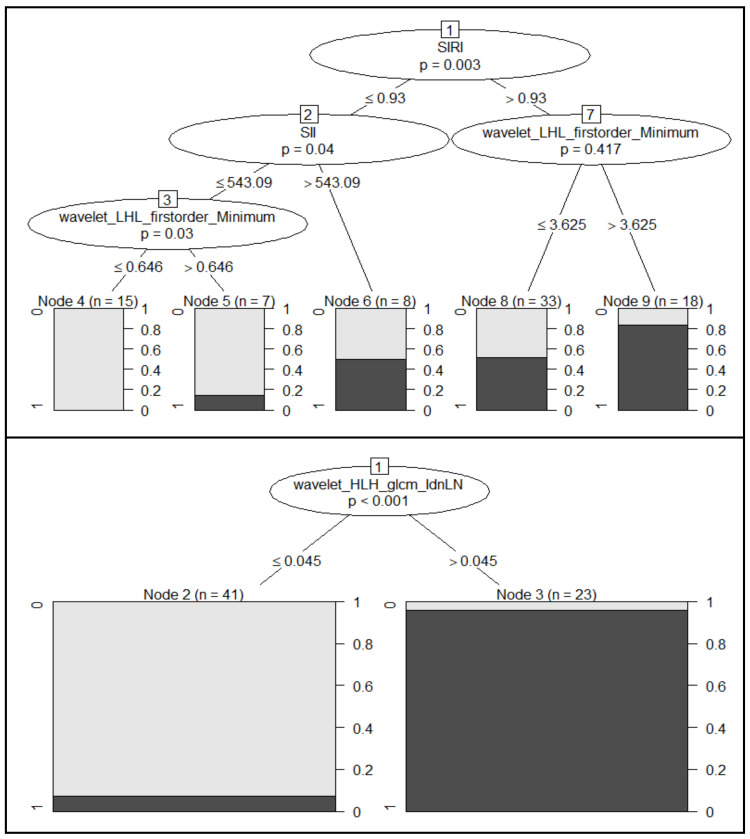
Decision trees obtained with the CIDT algorithm for the prediction of tumor grading (**top** panel) and metastatic lymph nodes (**bottom** panel). For each inner node, the Bonferroni-adjusted *p*-values are given, while the fraction of responsive patients is displayed for each terminal node.

Good performances were obtained when classifying patients according to tumor grading (G1 + G2 grading versus G3 grading). Specifically, radiomics features alone can predict tumor grading with an accuracy of 0.76 (sensitivity of 0.78, specificity of 0.75) and using a logistic regression model (Table 3). However, the predictive power of radiomics features can be further improved by combining them with clinical parameters. In fact, as shown in Table 3, the CART method achieved an accuracy of 0.82 (sensitivity of 0.78, specificity of 0.87) combining (left panel of Figure 1) three clinical parameters (SIRI, DOI, and PLR) with a radiomics feature extracted from tumor areas (*wavelet_LLL_glszm_SizeZoneNonUniformityNormalized*). This result was also confirmed by the CIDT method. In particular, despite the low accuracy value (0.65), it is possible to note that even CIDT (top panel of Figure 2) found the SIRI variable to be highly predictive (*p* = 0.003), with a cut-off value of 0.93.

Remarkable findings can be observed in the context of the prediction of metastatic lymph nodes. Considering a baseline accuracy of 0.94, using radiomics features in a logistic regression model (Table 4), both CART and CIDT were found to achieve an asymptotic accuracy value of 1.00. The results and decision charts obtained (Figure 1, right panel, and Figure 2, bottom panel) emphasize that metastatic lymph nodes can be predicted with only one radiomics feature. Specifically, CART used the *wavelet_HHH_glrlm_LowGrayLevelRunEmphasis* feature for classifying patients with or without metastatic lymph nodes, with a cut-off value of 0.41. On the other hand, the variable showing a significant conditionally dependence (*p* < 0.001) with the metastatic lymph nodes found using the CIDT algorithm was the *wavelet_HLH_glcm_*Idn (cut-off equal to 0.045).

Further analysis was also performed for the prediction of both perineural and vascular infiltration; however, as the machine learning approach did not improve the performance achieved in the univariate analysis, and considering that a more complex multivariate model would be useless, the results have not been reported or discussed.

## 4. Discussion

Currently, the management of the neck dissection in early-stage OTSCC remains a controversial topic, especially if evaluations are made in risk/benefit terms with respect to the possible oncological outcomes and the possible treatment morbidity.

Various options have been proposed for the early detection of lymph node metastases. In 2015, the Sentinel European Node Trial (SENT) assessed the identification of metastatic lymph nodes through the sentinel lymph node biopsy on a sample of 415 patients, and reported an overall sensitivity and negative predictive value of 86% and 95%, respectively [11]. Moreover, approaches based on artificial intelligence, machine learning, and radiomics metrics are largely reported in the literature in the context of oncological studies [12,13,14,15,16,17,18,19,20,21,22].

Different studies suggested several clinical parameters as predictive markers for the presence of occult laterocervical metastases; some of the most used are the degree of differentiation of the tumor, the depth of tumor invasion, the perineural invasion, the vascular invasion, and the intercourse neutrophils/lymphocytes. However, as already reported by Tsushima et al., these parameters are not always sufficient to identify patients with a high risk of occult metastatic lymph nodes, as there is a significant heterogeneity in the outcomes within single-stage groups [21].

In our study, we assessed the predictive power of both clinical parameters and radiomics features, considered both individually and in combination. The focus was the prediction of the risk of metastatic lymph nodes and the tumor grade related to OTSCC. Specifically, we considered, as clinical indicators, the most recent indices of inflammation (NLR, PLR, LMR, SII, and SIRI) and the DOI. In fact, it is now widely recognized that the inflammatory response plays a critical role in tumor promotion and progression, potentially influencing the survival outcomes [23,24,25,26,27,28,29,30,31,32,33,34].

Since the eighth edition of the AJCC staging system, DOI has been added to the TNM as a staging criterion of oral cancers [23]. According to the latest edition of TNM, a DOI higher than 5 mm allows for classifying the neoplasm as T2, even in the case of tumors smaller than 2 cm [24]. Furthermore, the National Comprehensive Cancer Network suggested one staging dissection of the neck when the DOI is greater than 4 mm. D’Cruz et al. [28] performed a randomized control study and demonstrated that staging neck dissection has a better prognosis compared to therapeutic neck dissection. 

However, the main limitation is the absence of a standardized definition of the DOI, leading to confusing the infiltration thickness with the DOI [25,26,27,28].

In our study, DOI alone achieved an accuracy of 70% in discriminating the tumor grading, with a cut-off of 5.43 mm; a cut-off of 4.76 mm, instead, was found to be able to detect metastatic lymph nodes with an accuracy of 79%.

Moreover, NLR has recently established itself as a leading index of inflammation, recognized as an independent prognostic factor in many solid tumor studies, including head and neck sites.

Assuming the effectiveness of NLR as an independent prognostic factor in head and neck tumors, for the first time, Abbate et al. demonstrated its correlation with occult lymph node metastases in early-stage carcinomas of the tongue [32]. The results of these authors demonstrated that the neutrophil/lymphocyte ratio was not only an independent prognostic factor for survival, but was is also useful for identifying patients at highest risk of occult metastatic lymph nodes.

This result was also confirmed by Wu YY et al. in their study on 890 patients with cancers of the oral cavity [34].

Furthermore, Kumarasamy C et al. [35] confirmed, with their systematic review of 34 studies, that PLR and LMR have been recognized as valid prognostic factors in the therapeutic management of oncological pathologies of the head and neck [24]. On the other hand, Feng Y [24] obtained significant prognostic efficacy with the use of SII and SIRI indices. In line with this, Valero C et al. [36] found that SIRI has a prognostic capacity in squamous-cell tumors of the head and neck area, noticing a significant decrease in survival for patients with higher values of SIRI.

These findings were confirmed by our results, with particular reference to the detection of metastatic lymph nodes. In fact, SII and SIRI can identify patients with metastatic lymph nodes with an accuracy of 78%, while NLR reported and accuracy of 79%. In addition, it was possible to detect metastatic lymph nodes with an accuracy of 83% using PLR alone. Similar results were also found in the classification of patients based on tumor grading (G1 + G2 versus G3 grading); in fact, PLR, SII, and SIRI were able to classifying the patients with an accuracy of 70%, when considered individually.

However, in the univariate analysis, no good performance was obtained by a single parameter.

Higher performances were achieved when considering radiomics metrics; the *wavelet_HHH_glrlm_LowGrayLevelRunEmphasis* feature, in fact, showed an accuracy of 96% in the identification of metastatic lymph nodes. The ability of a single radiomics predictor in identifying metastatic lymph nodes was confirmed in the multivariate analysis; using the CART algorithm, in fact, it was possible to classify metastatic and non-metastatic lymph nodes using only the *wavelet_HHH_glrlm_LowGrayLevelRunEmphasis* feature, reaching an asymptotic accuracy value of 100%. The cut-off value identified by the algorithm (0.41) was almost equal to that identified in the ROC analysis (0.39). Comparable results were observed when using a combination of 15 radiomics features in a logistic regression models with an accuracy of 94%. 

That a single radiomics feature is able to classify metastatic lymph nodes was also confirmed by the CIDT method, where the *wavelet_HLH_glcm_Idn* feature showed a high correlation with the outcome (asymptotic accuracy of 100%, *p* < 0.001).

Further good results were obtained for predicting the tumor grading with a combination of radiomics metrics and clinical parameters. The CART algorithm reached an accuracy of appropriately 82% when combining SIRI, DOI, and PLR with the *wavelet_LLL-glszm_SizeSoneNonUniformityNormalized* feature. In particular, it is interesting to make an observation regarding the SIRI variable and the cut-off values identified during the analyses related to tumor grading; in fact, the cut-off for the SIRI variable (0.95) identified by the CART method was almost equal to that identified in the ROC analysis (cut-off of 0.93) and with the CIDT method (cut-off of 0.93).

No significant results were, instead, found when predicting both the perineural and vascular infiltrations.

Given its preliminary nature, this study has some limitations. The manual segmentation of ROIs makes the data operator-dependent; this poses challenges in terms of study reproducibility. Moreover, despite the validation-based approach used in the multivariate analysis, the small sample size could introduce some bias in the results, although the cross-validation technique tends to mitigate this risk. Both these limits can be significantly reduced by increasing the number of samples and recruiting patients in additional medical centers. In this way, in fact, it is possible not only to obviate the limited training abilities of the machine learning algorithms due to the small sample size, but it is also possible to dilute the bias that affects the data, which is due to the manual segmentation of the radiologist. Semi-automated segmentation could also be considered in order to improve reproducibility of the results.

## 5. Conclusions

In conclusion, the results obtained in this preliminary study agree with the literature [37,38,39], and confirm that both radiomics features and clinical parameters have an important role in identifying tumor grading and metastatic lymph nodes. Machine learning approaches can be used as an easy-to-use tool to stratify patients with early-stage OTSCC, based on the identification of metastatic and non-metastatic lymph nodes. However, some limitations of this preliminary study need to be highlighted. This study has a retrospective nature and the segmentation of ROIs was manually performed by radiologists. Moreover, the small sample size certainly had an impact on the results discussed, especially in the context of the machine learning approach. Overcoming these limitations should be one of the main goals of further studies.

## Figures and Tables

**Table 1:** Univariate analysis. Results of the ROC analysis of the following clinical parameters: depth of invasion (DOI), neutrophils−lymphocytes ratio (NLR), platelet−lymphocyte ratio (PLR), lymphocytes−monocytes ratio (LMR), Systemic Inflammation Response Index (SIRI), Systemic Immune-inflammation Index (SII), and tumor largest diameter (SIZE).

**Tumor Grading**							
	DOI	NLR	PLR	LMR	SII	SIRI	SIZE
AUC	0.72	0.65	0.66	0.34	0.73	0.70	0.74
Sensitivity	0.68	0.89	0.51	0.97	0.68	0.86	1.00
Specificity	0.73	0.43	0.86	0.09	0.73	0.57	0.39
PPV	0.68	0.57	0.76	0.47	0.68	0.63	0.58
NPV	0.73	0.83	0.68	0.80	0.73	0.83	1.00
Accuracy	0.70	0.64	0.70	0.49	0.70	0.70	0.67
Cut-off	5.43	2.11	153.33	2.67	563.26	0.93	19.00
**Metastatic Lymph Nodes**							
	DOI	NLR	PLR	LMR	SII	SIRI	SIZE
AUC	0.82	0.73	0.76	0.22	0.72	0.74	0.70
Sensitivity	0.89	0.74	0.80	0.06	0.77	0.74	0.86
Specificity	0.72	0.83	0.85	0.96	0.78	0.80	0.57
PPV	0.70	0.76	0.80	0.50	0.73	0.74	0.60
NPV	0.89	0.81	0.85	0.57	0.82	0.80	0.84
Accuracy	0.79	0.79	0.83	0.57	0.78	0.78	0.69
Cut-off	4.76	2.92	142.02	7.47	563.26	1.42	23.00
**Perineural Infiltration**							
	DOI	NLR	PLR	LMR	SII	SIRI	SIZE
AUC	0.77	0.57	0.65	0.34	0.67	0.65	0.62
Sensitivity	0.74	0.69	0.59	0.03	0.69	0.64	0.36
Specificity	0.74	0.55	0.74	1.00	0.74	0.74	0.90
PPV	0.73	0.59	0.68	1.00	0.71	0.69	0.78
NPV	0.76	0.66	0.66	0.53	0.72	0.69	0.60
Accuracy	0.74	0.62	0.67	0.53	0.72	0.69	0.64
Cut-off	5.11	2.41	145.55	7.52	563.23	1.37	32.00
**Vascular Infiltration**							
	DOI	NLR	PLR	LMR	SII	SIRI	SIZE
AUC	0.69	0.64	0.53	0.40	0.64	0.65	0.58
Sensitivity	0.93	0.50	0.54	0.96	0.64	0.68	0.93
Specificity	0.49	0.85	0.62	0.08	0.72	0.70	0.25
PPV	0.49	0.64	0.43	0.36	0.55	0.54	0.39
NPV	0.93	0.76	0.72	0.80	0.79	0.80	0.87
Accuracy	0.64	0.73	0.59	0.38	0.69	0.69	0.48
Cut-off	3.64	3.26	142.02	2.67	578.01	1.42	18.00

**Table 2:** Univariate analysis. Most significant results of the ROC analysis of radiomics features extracted from ROIs on both the tumor and lymph node areas.

Performance Results	Tumor Gradin—Tumor Area	Metastatic Lymph Nodes—Lymph Node Area	Perineural Infiltration—Tumor Area	Vascular Infiltration—Tumor Area
	Original_Glszm_Highgraylevelzoneemphasis	Wavelet_HHH_Glrlm_Lowgraylevelrunemphasis	Wavelet_HHH_Glcm_Maximumprobability	Wavelet_LLL_Glszm_Highgraylevelzoneemphasis
AUC	0.66	0.93	0.65	0.62
Sensitivity	0.76	0.94	0.69	0.29
Specificity	0.66	0.98	0.67	0.98
PPV	0.65	0.97	0.66	0.89
NPV	0.76	0.96	0.70	0.72
Accuracy	0.70	0.96	0.68	0.74
Cut-off	−0.19	0.39	4.97	0.03

**Table 3:** Multivariate analysis. Results of machine learning approaches (logistic regression and tree-based algorithms) in the prediction of tumor grading.

Performance Results	Clinical Features	Radiomics Features	Combination of Both Clinical and Radiomics Features		
	Logistic Regression	Logistic Regression	Logistic Regression	CART	CIDT
Accuracy	0.65	0.76	0.59	0.82	0.65
Sensitivity	0.44	0.78	0.56	0.78	0.33
Specificity	0.87	0.75	0.62	0.87	1.00
No. of features	3	2	6	4	4

**Table 4:** Multivariate analysis. Results of machine learning approaches (logistic regression and tree-based algorithms) in the prediction of metastatic lymph nodes.

Performance Results	Clinical Features	Radiomics Features	Combination of Both Clinical and Radiomics Features		
	Logistic Regression	Logistic Regression	Logistic Regression	CART	CIDT
Accuracy	0.76	0.94	0.88	1.00	1.00
Sensitivity	0.57	1.00	0.86	1.00	1.00
Specificity	0.90	0.90	0.90	1.00	1.00
No. of features	4	15	20	1	1

## Data Availability

Data are available at link: https://zenodo.org/record/6361733#.YjM7z-rMK3A.
